# Monkeypox, a Literature Review: What Is New and Where Does This concerning Virus Come From?

**DOI:** 10.3390/v14091894

**Published:** 2022-08-27

**Authors:** Giorgio Tiecco, Melania Degli Antoni, Samuele Storti, Lina Rachele Tomasoni, Francesco Castelli, Eugenia Quiros-Roldan

**Affiliations:** 1 Department of Clinical and Experimental Sciences, Unit of Infectious and Tropical Diseases, University of Brescia and ASST Spedali Civili di Brescia, 25123 Brescia, Italy; 2 Unit of Infectious and Tropical Diseases, ASST Spedali Civili di Brescia, 25123 Brescia, Italy

**Keywords:** Monkeypox, MPX, MPXV, phylogenesis, vaccines, therapy

## Abstract

Among the *Poxviridae* family, *orthopoxvirus* is the most notorious genus. Several DNA viruses belonging to this group are known to produce human disease from the life-threatening variola virus (VARV) (the causative agent of smallpox), monkeypox virus (MPXV), cowpox virus (CPXV), and vaccinia virus (VACV). These *orthopoxviruses* still remain a public health concern as VACV or CPXV still cause emerging endemic threads, especially in developing countries. MPXV is able to cause sporadic human outbreaks of a smallpox-like zoonotic disease and, in May 2022, hundreds of cases related to MPXV have been reported from more than 30 countries around the globe. At the end of July, monkeypox (MPX) outbreak was even declared a global health emergency by the World Health Organization (WHO). Many aspects remain unclear regarding this outbreak and a deep understanding of *orthopoxvirus* might have crucial and evident implications. During the era in which people under 45 years old are not protected against VACV, the potential use of *orthopoxviruses* as a biological weapon raises global concern considering the rapid spreading of the current MPX outbreak in vulnerable populations. Hence, we review the most recent evidence about phylogenesis, pathogenesis, prevention, and treatment for this concerning disease.

## 1. Introduction

*Poxviruses* are the largest mammalian DNA viruses whose life cycle takes entirely place in the cellular cytoplasm [1]. These viruses encode a large set of proteins providing the extranuclear synthesis of viral mRNAs, replication of the genome, and assembly of the complex virions. These proteins are also involved in the regulation of multifactorial interactions with the infected host’s organism [1,2]. Among the *Poxviridae* family, *orthopoxvirus* is the most notorious genus. Several DNA viruses belonging to this group are known to produce human disease from the life-threatening variola virus (VARV) (the causative agent of smallpox), monkeypox virus (MPXV), cowpox virus (CPXV), and vaccinia virus (VACV) [2]. MPXV, CPXV, and VACV have a wide range of hosts, various rodents first and foremost, and humans only sporadically [2]. These *orthopoxviruses* are immunologically cross-reactive and cross-protective, so that infection with any member of this genus provides protection against an infection with any other member, and this property makes them very interesting for research [3]. *Orthopoxviruses* still remain a public health concern. VACV or CPXV still cause emerging endemic threads, especially in developing countries [4,5,6]. VARV, despite its eradication, is a priority of biodefense preparedness research [7]. MPXV, another *orthopoxvirus,* can cause sporadic human outbreaks of smallpox-like zoonotic disease [8]. In May 2022, hundreds of cases related to MPXV were reported from over 30 countries [2]. At the end of July, the monkeypox (MPX) outbreak was declared a global health emergency by the World Health Organization (WHO) [9]. Many aspects of these outbreaks remain unclear. A deeper understanding of *orthopoxvirus* is needed and might have crucial and evident implications. Available data demonstrates that vaccines based on VACV can induce cross-protective antibodies against other *orthopoxviruses* and might be used as vaccine backbone for recombinant vaccines [10]. At a time when people under 45 years old are not protected against VACV, the potential use of *orthopoxviruses* as biological weapons raises global concern. Below, we review the most recent evidence regarding phylogenesis, pathogenesis, prevention, and treatment available on this concerning disease.

## 2. Virology and Phylogenesis

Current data suggest that the natural MPXV lifecycle is a complex interaction of reservoir hosts and incidental species [11]. In 1958, MPXV was firstly isolated during a non-fatal outbreak in an animal facility from Asian monkeys used for polio vaccine research at the Statens Serum Institut in Copenhagen, Denmark [2]. A year later in Philadelphia, USA, a new MPX outbreak was reported in a colony of captive monkeys [12]. MPXV is capable of infecting and inducing disease in many animals within the *Mammalia* class; rope and tree squirrels, Gambian pouched rats, and dormice are other natural host of this virus that is then incidentally transmitted to humans when they encounter infected animals [2]. The first known human case of MPXV infection was recorded in 1970 in the Democratic Republic of Congo [2].

Several questions regarding the emergence of new threats to humankind caused by the evolution of *orthopoxviruses* remain open, but, thanks to phylogenetic analysis, it is possible to date back when and from whom the MPXV comes from. It was calculated that the *orthopoxviruses* first appeared approximately 40,000 years ago [8]. The ancestors of the so-called “modern species” originated between 1700 and 6000 years ago and, although VARV emerged around 300 AD, the separation of MPXV took place earlier, about 3500 years ago. It is estimated that MPXV first appeared in West Africa 600 years ago [8].

Although there is a common ancestor, the analysis of MPXV genome demonstrates that MPXV cannot be the direct ancestor or direct descendent of VARV [13]. Among its 196.858 base pair (bp) genome, similarly to other *orthopoxviruses*, each end of the genome contains an identical but inverted 6379 bp terminal repetition. However, comparative analysis within *orthopoxviruses* shows that MPXV poses about 190 open reading frames containing more than 60 amino acid residues, and in contrast with other viruses of the genus, only a limited subset of the putative immunomodulatory and host range genes has been identified in MPXV [13]. Thus, sequence comparisons make MPXV a distinct species, one perhaps evolved independently from a cowpox-like strain [13].

The evolutionary rate of the *orthopoxviruses* has previously been estimated to be between 10^−5^ and 10^−6^ mutations per replication site, translating into about 1–2 nucleotide changes per year for a nearly 200,000 bp genome [14]. The MPXV involved in the current outbreak differs by approximately 40 nucleotide mutations from the strains sequenced 4 years ago, suggesting an increase from 1 to 12 mutations per genome per year [15,16]. Since MPXV is considered a zoonotic virus with limited human-to-human transmission, the adaptation to humans may be the result of a long evolution leading to the sustained inter-human transmission that is now observed.

Before the MPXV whole genome sequencing, electron microscopy analysis of specimens from MPX rash was used to identify brick-like MPX virion that were indistinguishable from VARV or VACV virions [17]. In an effort to differentiate these *orthopoxvirus* species, infected embryonated hen eggs were used as the MPXV forms characteristic pock and genome restriction through endonuclease digestion. Two main mature virion shapes were observable: the M (mulberry) form with the characteristic mulberry protrusions and lack of phosphotungstic acid (PTA) that is present in vesicular fluid and isolated in crust specimens, and the C (capsule) form in which PTA penetrates the particle [17].

According to genome analysis, MPXV is conventionally classified into 2 different clades that diverged approximately 560–860 years ago in the African continent [18]. Perhaps, this specific period was characterized by environmental modification possibly creating the ecological conditions for the, at the time unknown, MPXV reservoir(s) to migrate [18]. The main genomic differences between the two clades are localized in the terminal regions that encode for host-response modifier proteins [11]. The first clade was isolated from West Africa (WA MPXV or Clade 2) and has experienced a limited drift [18]. It is less virulent as it contains deletions and fragmentations in the open reading frame [19]. This clade has a spatially structured sub-population located West and East of the Dahomey Gap [18].

The second historically known clade is endemic to Central Africa and, more precisely, to the Congo Basin (CB MPXV or clade 1) and has caused a more severe and more transmissible disease [2]. The CB MPXV downregulates the host responses; specifically, it prevents T-cell receptor-mediated T-cell activation [20]. In fact, a significant decrease in the T-cell-mediated cytokine production was observed [21]. This clade has likely experienced an expansion after a bottleneck or a founder effect/migration event. The four sub-populations identified do not show geographic structuring [18].

**Figure 1:**
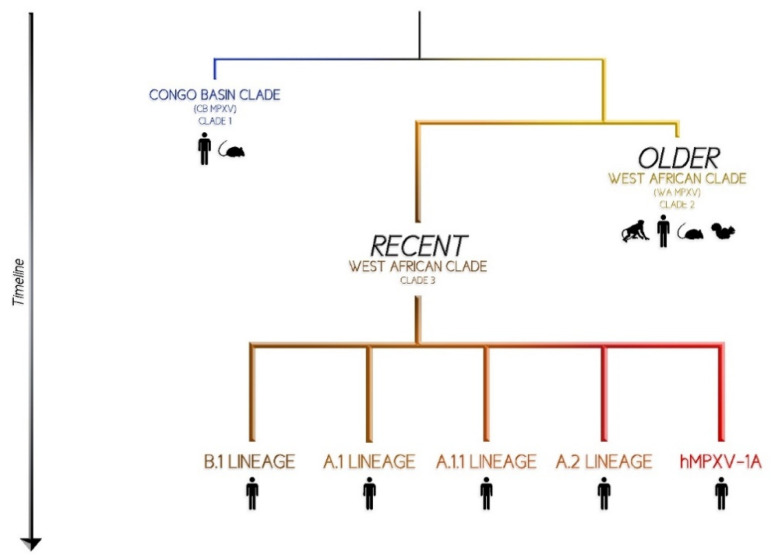
“Where does MPXV come from?” a graphical representation.

A more recent clade (MPXV Clade 3) including isolates originated during the 2017/2019 outbreaks in UK, Israel, Nigeria, USA, and Singapore, has been described and, together with clade 2, corresponds to the prior “West African clade” [22]. The first multi-country outbreak in non-endemic nations is currently underway, and it has raised an international public health emergency [9]. Phylogenomic analysis from the ongoing MPX outbreak confirms that the strain currently circulating descends from the clade 3 sampled in 2017/2019, which is similar to the one isolated in Nigeria [22]. It is also included within the formerly designated ‘West African’ clade (WA MPXV) (concretely within the clade 3) with a high number of mutations that raises concern about the increased capacity of adapting to humans [23] (Figure 1).

Comparison between 2022 MPXV genomes and the 2017/2019 outbreaks identified 47 shared single nucleotide differences, an unexpectedly large number in a short space of time [24]. Some authors have proposed a new and more convenient name for the virus causing this epidemic: “hMPXV1” has been suggested to denote where this now human virus becomes distinct from MPX. The hMPXV1 sub-clade presents a notable diversity even among the limited number of genomes so far described [22]. The same authors suggest a system similar to Pango nomenclature for SARS-CoV-2 with lineages to describe genealogical relationships [25]. Under this nomenclature, the base of hMPXV1 would be named lineage ‘A’, the descendant lineages ‘A.1’, ‘A.2’, ‘A.1.1’, and the current international 2022 outbreak ‘B.1’ [26].

## 3. Epidemiology in Humans

It should be noted that the diversity and extent of the animal reservoir are still unknown [27]. An increasing synanthropic rodent population, the subsequent rise of human-rodent interaction, together with a larger and more interconnected immune-naïve population, a result of the vaccination programs ending over 40 years ago, might explain the increased transmission of MPXV to humans [27]. Prior to 2022, MPX human cases were rarely reported outside Africa [2]. The major reported MPXV outbreaks occurred in 1970, 1996–1997, 2003, and 2018 [26]. The only human cases reported before 2018 happened almost exclusively in Nigeria and especially among gay-bisexual men (gbMSM) [28]. Of interest, during the 2003 outbreak in the US, 71 human cases derived from a shipment of infected Gambian pouched rats which subsequently infected prairie dogs [29]. Since early May 2022, monkeypox cases have been reported from multiple countries where the disease is not endemic.

At the time of writing, the reported cases globally, according to CDC, are 28,220, of which 27,875 in countries that have not historically reported monkeypox. According to ECDC, since the start of the monkeypox outbreak and as of 4 August 2022, 13 022 confirmed cases of monkeypox (MPX) have been reported from 28 EU/EEA countries [30,31].

In the 80s, mathematical models based on a reproductive index (R_0_) of 0.83 and under conditions of complete absence of vaccine-induced immunity suggested self-terminating outbreaks [32], but looking at the current epidemiological data, this assumption has to be reconsidered [27,32]. New mathematical models recalculated the reproductive index value of MPX and estimated an R_0_ between 1.1 and 2.4 during the current outbreak [19].

## 4. Transmission Route and Pathogenesis

As mentioned before, although the MPXV reservoir remains unknown, the MPXV infection affects several different animals, with both monkeys and humans being accidental hosts [33]. One of the routes of transmission is contact with an infected animal’s body fluid, through an animal bite or through the consumption of raw or minimally processed meat [34]. The intensity of animal contact correlates to the severity of clinical manifestations. The so-called “complex” exposure, defined as an invasive bite or scratch from an infected animal, was linked to pronounced signs of systemic illness, shorter incubation periods and, of course, an increased hospitalization rate [34].

MPX human-to-human transmission has been attributed to direct and intimate contact with infectious sores, scabs, or body fluids of an infected individual [35]. During the current outbreak, contact with the infectious viral material from skin lesions occurring during sexual intercourse has been identified as the main risk factor [36]. This is in line with the majority of the reported cases having no travel-related link to an endemic country [2]. Although it has been described especially among gbMSM, MPX is not a typical sexually transmitted disease (STD) [2]. It is still unknown whether MPX can spread through sexual bodily fluids; viral DNA has been recently detected in semen [37]. Perhaps, an STD-like outbreak might fill up the concerning lack of awareness about several at risk sexual practices in order to promote a better knowledge of STDs prevention, especially among adolescents and young adults [38].

The last direct transmission route is the mother-to-foetus route, as MPXV might cross the placenta. Congenital MPX has been described and concerning data have been reported in case series analysis [33]. For instance, in the Democratic Republic of the Congo, a report on four pregnant women infected by MPXV found that only one infant was born healthy [39]. Although the smallpox vaccine is still not recommended for pregnant woman, it is desirable that infants should at least promptly receive the smallpox vaccination in endemic areas of Africa. Additional research must be conducted to assess the effects of MPX on pregnant women [40].

Indirect transmission through infected fomites has been reported, for instance clothing or linens contaminated with infectious material from body fluids or sores [35]. The case of imported MPX in a traveler returning from Nigeria was anecdotal, as environmental analysis confirmed viral isolation from porous and non-porous surfaces, suggesting the need of additional cleaning in locations where MPX-infected patients have stayed [41]. Furthermore, MPX might spread through respiratory secretions, but a prolonged face-to-face contact seems to be necessary [42]. Although far less than that observed in human smallpox, the transmissibility of the virus among unvaccinated individuals seems to have a considerable attack rate just within households [43].

Viral shedding and period of infectiousness are far from cleared. Every person is considered infectious from the onset of symptoms to the disappearance of skin lesions and complete re-epithelization. In terms of single-room isolation or cohortization, this indication is currently followed by clinicians in MPX-confirmed cases. However, sero-epidemiological analysis has revealed that asymptomatic or subclinical MPX infections do exist, and no data support or reject the hypothesis that these patients are able to transmit the infection [44]. Polymerase chain reaction (PCR) tests performed on respiratory tract samples and blood of several MPX-infected patients revealed a persistence of positivity up to 3 weeks, but, again, no correlation between this result and infectivity was hypothesized [45].

It is logical to think that several of the features described were already present in previously known MPX strains. Therefore, it is hard to understand the current exceptional surge of cases and the broader geographical expansion. According to a recent study, the driver of the current outbreak is not ascribable to the genetics of currently spreading lineages, but to multifactorial elements [26]. These might include the cessation of smallpox vaccination and globalization, which has changed people’s lifestyles and increased travel rates [26].

## 5. Clinical Manifestations

Generally, the clinical presentation resembles that of smallpox, but MPX has a specific distinguishing feature: the lymphadenitis, especially in sub-mental, submandibular, cervical, and inguinal regions [46]. People infected with MPXV usually present a mild disease; however, in certain situations, such as pregnancy or immunodeficiency, MPXV can cause severe disease [47].

Clinical manifestations of MPX infection usually appear after an incubation period of 5 to 21 days, and it is usually a self-limited disease [33,48]. MPX infection is mainly divided in two phases: the prodromal phase (lasting about 0–3 days) with fever, lymph node swelling, exhaustion, headache, chills, back pain, and muscle aches; and the rash phase (lasting 7–21 days) [49,50,51]. It is common, within three days after the onset of prodrome symptoms, for a centrifugal maculopapular rash to start from the site of primary infection and rapidly spread to other parts of the body. The rash is typically concentrated on the face and extremities, affecting the face (>90%), palms and soles of the feet (75%), oral mucosa (70%), genitals (30%), and conjunctiva (20%). The lesions progress, usually within 10 days and simultaneously from the stage of macules to papules, vesicles, pustules, crusts, and scabs, before falling off [47,49,51,52,53]. The published literature is lacking regarding laboratory findings. It is known that in smallpox, haematologic abnormalities such as lymphocytosis and thrombocytopenia have been observed early in severe confluent forms of infection. Similarly, clinical manifestations previously described, together with hematologic or hepatic laboratory abnormalities should induce a prompt inclusion of MPX in the differential diagnosis [54].

Rarely, human MPX cases may experience more severe symptoms: complications in endemic countries include encephalitis, secondary skin bacterial infections, dehydration, conjunctivitis, keratitis, and pneumonia [34,51]. Although the mortality rate in several previous human MPX outbreaks in Central Africa has reached 10.6% [55], the case fatality ratio of the current outbreak ranges between 3 and 6%. Between 1 January to 1 May 2022, the WHO reported 67 deaths in Democratic Republic of the Congo, 1 in Nigeria and only 3 cases outside Africa (1 in Brazil and 2 in Spain) [56]. It is important to consider the neuropsychiatric symptoms of MPX. There is preliminary evidence for a range of neurological and psychiatric presentations of MPX (from nonspecific neurological symptoms such as myalgia and headache to rarer but more severe neurological complications such as encephalitis and seizures). There is less evidence on the psychiatric sequelae and MPX-related nervous system presentations that may warrant surveillance within the current MPX outbreak [57,58]. Unfortunately, the psychological impact of MPX is not as well defined as it should, as the WHO recently called for greater attention to be paid to mental health problems and suicide prevention during epidemics [59].

The current outbreak is exposing gaps in our knowledge of MPX, and clinicians should be aware of the atypical manifestations of the disease. For instance, in some cases, both the absence of prodromal symptoms and the presence of herald skin lesions only at the point of sexual contact were observed [37,60,61]. It suggests that human-to-human transmission through close physical contact in sexual networks plays a key role in the current outbreak. Furthermore, in some cases, the synchronous evolution of the lesions does not seem to be certain, with asynchronous skin lesions manifesting instead [37,61]. An international collaboration across 16 countries reported 528 infections diagnosed between 27 April and 24 June 2022 [62,63]. In this study, 95% of these patients presented with a rash, 73% had anogenital lesions, and 41% had mucosal lesions. About 54 (10%) had only a single genital lesion. This is consistent with another recent observational analysis in UK that showed that all individuals with a confirmed MPX diagnosis were gbMSM, with a high proportion of concomitant sexually transmitted diseases and frequent anogenital symptoms. This suggests transmissibility through local inoculation during close skin-to-skin or mucosal contact [46].

The current international case definition needs to be expanded accordingly, including these atypical presentations in order to reduce misdiagnoses [62,63].

There are limited data about the MPXV/HIV coinfection. Previous studies in Africa stated that people with untreated HIV infection had more extensive and longer-lasting lesions, more complications, and an overall worse outcome [64]. As of today, there are only a few case reports or case series, but a recent analysis highlighted that in this coinfection, a pool of atypical manifestations might be present, for instance whitish papules in a kissing lesion configuration in the perianal area [65]. The largest study of confirmed MPX cases to date found that HIV infection was not linked to monkeypox severity [62].

## 6. Diagnosis

As the clinical manifestations of MPXV infection are difficult to distinguish from other *orthopoxviruses*-caused diseases, rapid diagnosis plays an essential role in controlling actual outbreaks. MPXV real-time polymerase chain reaction (real-time PCR) on suspected skin lesions is the preferred method for routine diagnosis [47,53]. Scabs, swabs, and aspirated lesion fluid are preferable over blood samples, due to limited duration of viraemia. The conserved regions of extracellular envelope protein gene (B6R), DNA polymerase gene E9L, DNA-dependent RNA polymerase subunit 18 (RPO18) gene, and complement binding protein C3L, F3L, and N3R genes are usually selected as targets for PCR amplification [49,66,67,68,69]. Certain real-time PCR assays can discriminate not only monkeypox virus from other *orthopoxviruses*, but also between the two MPXV clades observed [53].

Viral isolation and culture can also be required to establish a definitive diagnosis and immunochemistry analysis, and multiplexed immunofluorescence imaging could be used for monkeypox antigen detection [70]. Enzyme-linked immunosorbent assay (ELISA) can be used to detect the specific IgM and IgG antibodies in the serum of monkeypox patients after 5 and 8 days of infection, respectively. Serology has limited value due to the immunological cross-reactivity between human-pathogenic *orthopoxviruses*, although it can be useful for excluding a recent *orthopoxvirus* infection (for example, contact investigations and population serosurveys) [47,71].

## 7. Prevention

Two vaccines are currently available and licensed for smallpox, JYNNEOS (live, attenuated, replication incompetent vaccinia virus, two subcutaneous doses 28 days apart) and ACAM2000 (live, replication competent vaccinia virus, single-time administration percutaneously through scarification). These smallpox vaccines are thought to be about 85% effective against monkeypox infection, according to the CDC and the WHO [72].

These two vaccines are substantially different. Because ACAM2000 is replication-competent, there is a risk for serious adverse events (such as progressive vaccinia and eczema vaccinatum; myopericarditis and post-vaccine encephalitis also occur but the underlying mechanism is still unknown). JYNNEOS has fewer contraindications, has no risk for inadvertent inoculation and auto-inoculation, and a more comfortable administration. Moreover, JYNNEOS involves 2 vaccine doses 28 days apart and vaccine protection is not conferred until 2 weeks after receipt of the second dose; ACAM2000 involves 1 dose of vaccine and peak vaccine protection is conferred within 28 days [73,74]. In regard to the most fragile categories, JYNNEOS is safe to administer to persons with immunocompromising conditions, and it could be considered safe in pregnant women (animal models, including rats and rabbits, have shown no evidence of harm to a developing foetus) [75,76,77].

- Pre-exposure Prophylaxis

The Advisory Committee and Immunization Practices (ACIP) recommends vaccination for select persons at risk for occupational exposure to *orthopoxviruses* (research and clinical laboratory personnel, clinical testing for *orthopoxviruses*, designated response team members at risk for occupational exposure) [78]. There are no data regarding pre-exposure prophylaxis in the current outbreak.

- Post-exposure Prophylaxis

The CDC has recently developed informed guidance to assess the risk of exposures and make informed decisions about post-exposure prophylaxis. In particular, people at “high” or “intermediate” exposure risk (defined as a person who had “unprotected contact” with the skin or bodily fluids of someone with monkeypox, or who was within 1.8 meters, or 6 feet of an infected person) may have access to vaccination within 4 days of exposure (if given 4–14 days after contact, vaccination may reduce symptoms but not prevent disease onset) [72].

Countries including Canada, the United Kingdom, and the United States have begun implementing a strategy called ring vaccination to try and halt the spread of the virus [79]. At the moment, the risk posed by monkeypox to the general public is not high enough to warrant mass vaccination, and there are limited real-life data to support this guidance. Limited testing against monkeypox may lead to further issues (for example, it is unknown whether a single dose of JYNNEOS would suffice to stop the infection), difficulties related to the strait contact tracing needed, and vaccines-related side effects [80].

## 8. Treatment

As previously mentioned, most cases of monkeypox have mild and self-limited diseases; supportive care is typically sufficient without any medical treatment [48,73,81]. However, the prognosis for monkeypox may depend on multiple factors, such as initial clinical presentation, comorbidities, and previous vaccination status [48].

Several categories of patients should be considered for treatment, such as people with a severe disease (haemorrhagic disease, confluent lesions, sepsis, encephalitis, or other conditions requiring hospitalization); people who may be at high risk of severe disease (immunocompromised, paediatric populations, people with severe skin conditions, pregnant or breastfeeding women); people with one or more complications (secondary bacterial skin infection; gastroenteritis with severe nausea/vomiting, diarrhoea, or dehydration; bronchopneumonia; concurrent disease or other comorbidities); people with monkeypox virus aberrant infections that include accidental implantation in eyes, mouth, or other anatomical areas where monkeypox virus infection might constitute a special hazard (e.g., the genitals or anus) [48].

- Tecovirimat

Tecovirimat works by inhibiting the viral envelope protein VP37, which blocks the final steps in viral maturation and release from the infected cell, thus inhibiting the spread of the virus within an infected host [82,83]. It is the first antiviral indicated for the treatment of smallpox in adults and paediatric patients, and it is considered the treatment of choice [73,84]. Tecovirimat is available as a pill or injection.

In vitro studies confirmed that tecovirimat inhibited the replication of multiple *orthopoxviruses* in cell culture, demonstrating broad spectrum protective efficacy in multiple lethal animal models of *orthopoxvirus* disease (including *orthoopoxviruses* known to be human pathogens) [84,85].

In animal models, earlier initiation of treatment correlated with increased survival and a reduction in signs of illness. In studies where treatment was initiated one day following challenge, animals showed negligible signs of illness, suggesting that tecovirimat has potential not only for treatment but also for post-exposure prophylaxis [86].

Real-life data are still limited. A case series of individuals infected with MPXV in the UK and USA, including patients treated with tecovirimat, suggests that tecovirimat may shorten the duration of illness and viral shedding [45,79]. In another case report, an American traveler returning from Nigeria with severe illness was treated with tecovirimat, achieving a good clinical course [87].

However, its use for other *orthopoxvirus* infections, including MPX, is not approved by the FDA. Therefore, CDC holds a non-research expanded access Investigational New Drug (EA-IND) protocol that allows the use of tecovirimat for primary or early empiric treatment of non-variola *orthopoxvirus* infections, including monkeypox, in adults and children of all ages [48].

- Brincidofovir and Cidofovir

Brincidofovir (viral DNA polymerase inhibitor, analogue of the intravenous drug cidofovir) is an antiviral medication that was approved by the FDA in June 2021 for the treatment of human smallpox disease in adult and paediatric patients, including neonates. It has shown to be effective against *orthopoxviruses* in vitro and in animal studies [88]. Liver function tests before and during treatment must be done, as brincidofovir may cause an increase in serum transaminases and serum bilirubin, as observed in a UK case series of monkeypox infection [45].

- Vaccinia immune globulin intravenous (VIGIV)

VIGIV is licensed by the FDA for the treatment of complications due to vaccinia vaccination, including eczema vaccinatum, progressive vaccinia, severe generalized vaccinia, vaccinia infections in individuals who have skin conditions, and aberrant infections induced by vaccinia virus. CDC holds an expanded access protocol that allows the use of VIGIV for the treatment of *orthopoxviruses*, including monkeypox, even if there are no available data. VIGIV can be considered for prophylactic use in an exposed person with severe immunodeficiency in T-cell function, for which smallpox vaccination following exposure to monkeypox virus is contraindicated [48].

Currently ongoing clinical trials are described in Table 1 [89].

## 9. Conclusions

After more than two years of SARS-CoV-2 pandemic, it is understandable that the news of a new virus spreading across the globe could cause alarm. According to health experts, MPXV is unlikely to create a new pandemic. Longstanding weaknesses in the public health system are giving MPXV a chance to expand, despite MPXV not spreading efficiently and diagnostic tests and vaccines being available even before the current outbreak. As with SARS-CoV-2, mutations and natural selection have likely increased the transmissibility of MPVX. What we have learned from the COVID-19 pandemic can surely be useful to respond effectively to the MPXV outbreak with policymakers, researchers, and healthcare workers working together.

## 10. Research Strategy and Selection Criteria

References for this review were identified from PubMed, Embase, and Cochrane with the following research terms: “Monkeypox”, “MPX”, and “monkeypox outbreak”. These keywords were combined with “mutations”, “diagnosis”, and “treatment”. Only papers in English were included. This is a non-systematic review, and the final reference list was generated based on timeline, originality, and relevance to the scope of this review.

## Figures and Tables

**Table 1:** Currently ongoing clinical trials for MPX Treatment.

n. Trial and Title	Type	Description
**NCT05443867** Monkeypox Asymptomatic Shedding: Evaluation by Self-Sampling MPX-ASSESS	observational cohort prospective	Close follow-up study of close contacts of MPX confirmed cases. Evaluation of secondary attack rate of MPXV infection in contacts, defined by PCR positivity on any sample; clinical and serological evaluation.
**NCT05058898** A One Health Study of Monkeypox Human Infection	observational case-control prospective	Proportion of monkeypox cases occurring following interhuman exposures through a quantitative case-control study with an odds ratio of >3 for an exposure factor for human-to-human transmission.
**NCT05438953** Follow-up of Contact at Risk of Monkeypox Infection: a Prospective Cohort Study	Prospective Interventional	Estimation the failure rate of a post-exposure vaccination by the VMA vaccine in MPX contact case participants at risk (within 14 days after the last contact) after one dose.
**NCT02977715** IMVAMUNE^®^ Smallpox Vaccine in Adult Healthcare Personnel at Risk for Monkeypox in the Democratic Republic of the Congo	prospective interventional	Description of monkeypox exposure and infection in eligible healthcare workers at risk of monkeypox infection, evaluating the immunogenicity and safety MVA vaccine. Evaluation of proportion of participants who after being vaccinated develop suspected or confirmed monkeypox infection, and experience exposure to monkeypox virus.
**NCT02080767** Tecovirimat (ST-246) Treatment for Orthopox Virus Exposure	prospective interventional	Evaluation of efficacy and safety of tecovirimat in personnel (including US civilian employees, contractors and other US personnel and dependents, as well as allied military forces and local nationals) of any age exposed to or infected with *orthopoxviruses* or developed serious complications from vaccinia vaccination.

## Data Availability

Non applicable.
